# Our Hospitals and the National Relief Fund

**Published:** 1916-08-19

**Authors:** 


### OUR HOSPITALS AND THE NATIONAL RELIEF FUND.

### THE POINT OF VIEW OF THE SPECIAL HOSPITALS.

The position of the special hospital at the commencement of the war was a difficult one. The temptation in all cases to offer beds to the War Office for the use of the wounded, and thus to secure revenue, not only from a capitation grant, but from new supporters on what has been proved to be a very successful ground for appeal, was strong indeed. The alternative was to proceed on the old lines, knowing that as many beds were withdrawn from civilian use at the general hospitals the calls upon the accommodation of the special hospitals would become more insistent. Happy was the hospital which could conscientiously lend the use of its organisation to the country within the limits of its own speciality; but these cases were few, and while the ophthalmic hospital could help numbers of recruits to pass the medical tests, and the hospitals for nervous diseases could deal with cases of severe shock and nerve injury coming from abroad, there were many institutions, such as those for women and children, which, should they join in augmenting the resources of the military, could only do so by throwing their charters overboard.

If a distribution on the part of the National Relief Fund should be made amongst hospitals it seems reasonable to expect that these special hospitals, notably hospitals for women and children, which have contented themselves by quietly labouring within their own sphere, and have put on one side tempting opportunities of sharing in the public support of "war work," shall have special consideration. Their need in many instances is at the present time very great; from a large number of reports we have had under review showing the figures for 1915 it is learned that for every institution of this class which had a surplus on the year's working there were three which had a deficit, while amongst the hospitals which lent beds to the War Office and received the accruing advantages a prosperous income and expenditure account is a rule rather than an exception.

Of the institutions referred to, some examples may be cited. The British Royal Hospital for Women and Children has a waiting list of eleven, in spite of the fact that the number of patients nursed last year exceeded by about two hundred that of the previous year. While the expenditure has considerably increased, the income for the last three years has remained at the same level. The Queen's Hospital for Children, Hackney Road, has a waiting list of sixty, although large numbers of urgent cases have had to be referred to other institutions and the official number of beds is considerably increased by the use of balconies. The income has not increased, and at the present time there is an overdraft at the bank of over £4,000. The Royal London Ophthalmic Hospital, City Road, has devoted a few beds to soldiers suffering from diseases and injuries of the eye, and has treated a large number of soldiers and potential soldiers in the outpatient department. There is a waiting list of eighty-four civilians, and the estimated income for 1916 is lower than both 1914 and 1915. The Royal Waterloo Hospital for Children and Women also shows a falling income and a heavy waiting list (eighty-seven women and thirty-three children).

### Metropolitan Hospitals.

We have received numerous letters on this subject, from which we select the following:—

### Guy's Hospital.

I enclose herewith particulars of our income from voluntary sources for the past two years, and for the current year so far as it is possible to give any estimate. The number of patients on our waiting list is also included. We cannot separate the number of women and children, as the children's cots are in the women's wards, and we keep no separate record.—W. J. Curry, Clerk.

*Income from Voluntary Sources.*

| | 1914 | 1915 | Estimate. 1916 |
|---|---|---|---|
| Annual subscriptions ... | 3,341 | 3,247 | 3,000 |
| Donations ... ... ... | 2,841 | 5,671 | 5,500 |
| King Edward's Hospital Fund ... ... ... | 6,500 | 6,000 | — |
| Hospital Sunday Fund ... | 1,692 | 2,022 | — |
| Hospital Saturday Fund ... | 317 | 336 | 414 |
| Congregational collections | 8 | 16 | — |
| Workmen's collections ... | 98 | 161 | 100 |
| Legacies ... ... ... | 29,447 | 7,339 | 535 to date. |
| Total | £44,244 | £24,792 | — |

Patients on waiting list, August 9, 1916: Men, 260; women and children, 453; total, 713.

### Westminster Hospital.

Mr. Sydney M. Quennell writes: I am sorry that I cannot give you in detail the number of applicants on our waiting lists for in-patient treatment, but I can safely say that it is much larger than in normal times, and would be larger still if we felt it fair to book the names of those with regard to whom there is no probability of our being able to admit within a reasonable period.

Similarly with regard to finance, it is very difficult to forecast what our receipts are likely to be for the year ending December 31, 1916, but I can say as regards the six months ended June 30 last that the receipts were £10,989 (including £3,815 from legacies), which in ordinary times would have been sufficient to cover the expenditure for the same period, but the actual fact is that it failed to meet the expenditure by nearly £2,000, this being due to the ever-increasing cost of all commodities and to the heavy payments that it is now necessary to make in order to secure the services of a staff of resident medical officers even below the ordinary number.

THE NATIONAL RELIEF FUND—(*Continued.*)

### PROVINCIAL HOSPITAL MANAGERS' VIEWS.

### Manchester Royal Infirmary.

The Manchester Royal Infirmary will not stand alone in showing a reduction in the number of patients awaiting admission. The *working* habit has gripped the medical profession no less than any other class of workers. One of the grandest features of the past two years has been the non-curtailment of the hospital privileges enjoyed by the civil population, and that, in spite of the thousands of military patients who have passed through the civil hospitals. If a comparison is made between the medical (as opposed to the surgical) patients admitted since August 1, 1914, and the two previous years, a very marked decrease will be shown. The absence of so many men with the Army has created a smaller demand for male surgical admission, and consequently has increased the number of beds available, which have been used for those awaiting admission.

The cause of the general hospital cannot be pleaded on an increased waiting list, but it can be claimed that in spite of the Central Medical War Committee claiming the services of those best fitted to cope with night-and-day work; in spite of the nursing associations claiming the certificated nurses; and in spite of the King and country claiming all the remaining able-bodied members of its staff, the general hospital has fought hard for its country, adding a bed wherever a bed could be added, even (to quote the case of the Manchester Royal Infirmary) to 425 beds, without curtailing the privileges of the civil population, as is shown by its diminishd list of those awaiting admission. —Frank G. Hazell.

*Revenue from Voluntary Sources.*

| | 1914 | 1915 | 1916 (estimated) |
|---|---|---|---|
| Annual subscriptions ... | 11,509 | 11,925 | 12,000 |
| Donations ... ... ... | 1,726 | 1,706 | 1,750 |
| Alexandra Day ... ... | 731 | 892 | 600 |
| Hospital Sunday Fund ... | 1,036 | 880 | 1,142 |
| Hospital Saturday Fund ... | 807 | 812 | 855 |
| Congregational collections | 107 | 100 | 100 |
| Workmen's collections ... | 67 | 123 | 190 |
| In-patients' contributions | 4,403 | 4,328 | 3,465 |
| Out-patients' contributions | 663 | 620 | 691 |
| Total | £21,049 | £21,386 | £20,793 |

The number of operations performed during the first seven months of each of these years has a distinct bearing on the waiting list, and will therefore be of interest.

Operations performed between January 1 and August 1: 1914, 4,347; 1915, 4,946; 1916, 4,998.

The waiting list on August 1: 1914, 498; 1915, 830; 1916, 171.

The waiting list on the 1st instant comprised 41 males, 129 females, and one child, a total of 171.

### The Royal Infirmary, Sheffield.

If hospitals generally are to be considered, the claims of the Royal Infirmary, Sheffield, should receive *special consideration*. The infirmary has now 374 beds, 133 of which are set apart for wounded soldiers, and, on account of the special facilities afforded, many of the worst cases coming into Sheffield are treated here. Up to the present time nearly 1,500 military cases have been treated as in-patients, some for very long periods—in a few instances men have been patients for more than a year. The *x*-ray and massage departments have been enlarged to deal with the increased work. Unlike most hospitals, the Royal Infirmary Board have asked for no payment whatever from the State for this colossal work, the wish of the board being, in a crisis like the present, to assist the Government in every possible way.

It is estimated that the number of in-patients treated for the year ending December 31, 1916, will exceed 5,000, and that the out-patients will in the same period total 40,000. The annual expenditure in 1915 was £23,594, and owing to the alarming advance in the price of nearly every commodity it is expected that the total annual expenditure will in 1916 approach £27,000. The total revenue, exclusive of legacies, from voluntary sources in the years 1914 and 1915, and the approximate voluntary revenue for 1916, are as follows:—1914, £13,207; 1915, £16,663; 1916, £15,878. Our waiting list on August 8, 1916, contained 871 names. These were made up as follows:—Male adults, 674; female adults, 133; children, 64. The infirmary has supplied a very large number of doctors and nurses to one or other of the national Services.—Jno. W. Barnes, Secretary.

### County Hospital, Lincoln.

I beg to inform you that at the moment we have only seven patients on our waiting list. This number is far smaller than usual, but we have noticed that for some months past applicants for admission to the hospital have been fewer than usual, although the population of the town is largely increased. Our income from voluntary sources for two years past, and an estimate of the income from such sources for the current year has been: Voluntary income, 1914, £4,314; 1915, £4,209; 1916, £4,010.

Owing to the increased cost of all commodities our expenditure has increased heavily, and, in spite of money received for the treatment of soldiers in our wards, we fear that by the end of the year we shall be faced with a deficiency of between £1,500 and £2,000.—Arthur Moore, Secretary-Superintendent.

### AGAINST ANY GRANT FROM THE RELIEF FUND.

Referring to your article on the subject of our "Hospitals and National Relief Fund," if the Fund is used as generously as intended for the purpose for which I believe it was given, there will be no surplus for distribution amongst the hospitals. I do not think the hospitals are entitled to it, and should certainly not be a party to asking for any portion of the amount.—Superintendent.

